# Human Milk Macronutrient and Energy Contents Are Associated with Maternal and Infant Factors: A Cross-Sectional Analysis of Data from the Japanese Human Milk Study Cohort

**DOI:** 10.1016/j.cdnut.2025.107579

**Published:** 2025-10-17

**Authors:** Keisuke Nojiri, Kyoko Nomura, Tomoki Takahashi, Yuta Tsujimori, Takehiko Yasueda, Satoshi Higurashi

**Affiliations:** 1 Research and Development Department, Bean Stalk Snow Co., Ltd., Kawagoe, Japan; 2 Department of Environmental Health Science and Public Health, Akita University Graduate School of Medicine, Akita, Japan

**Keywords:** human milk, protein, carbohydrate, fat, energy, Japan, exclusive breastfeeding, obesity, birth weight, days postpartum

## Abstract

**Background:**

Human milk (HM) macronutrients are vital for infant growth and development; their composition may vary according to maternal and infant characteristics and differ across populations. However, the wide range of maternal and infant factors affecting HM macronutrient content among Japanese mothers has not been sufficiently evaluated.

**Objectives:**

We comprehensively examined the factors associated with the macronutrient content of mature HM in a cross-sectional analysis of a large Japanese cohort.

**Methods:**

A cross-sectional analysis was conducted using baseline data from the Japanese HM Study cohort (*n* = 1071). Macronutrient and energy contents were assessed in mature HM samples collected at an average of 2 mo postpartum. Maternal and infant factors were obtained via self-reported questionnaires. Linear regression analyses were performed to identify associations between these factors and HM macronutrient and energy contents.

**Results:**

Multivariable linear regression revealed significant associations between HM composition and several maternal and infant factors. True protein content was negatively associated with days postpartum [days; partial regression coefficient (*B*) = −0.0036, *P* < 0.001] and exclusive breastfeeding (*B* = −0.1045, *P* < 0.001). Carbohydrate content was positively associated with exclusive breastfeeding (*B* = 0.0963, *P* < 0.001). Fat content had a positive association with maternal overweight or obesity (*B* = 0.4648, *P* = 0.008) and negative associations with birth weight (g; *B* = −0.0002, *P* = 0.043) and days postpartum (days; *B* = −0.0103, *P* < 0.001). Crude protein and energy contents showed patterns similar to those of true protein and fat, respectively.

**Conclusions:**

For Japanese mothers, the macronutrient and energy contents of mature HM were associated with exclusive breastfeeding, maternal overweight or obesity, infant birth weight, and days postpartum.

This trial was registered at https://center6.umin.ac.jp/cgi-open-bin/icdr_e/ctr_view.cgi?recptno=R000017649 as UMIN000015494.

## Graphical abstract

**Figure:**
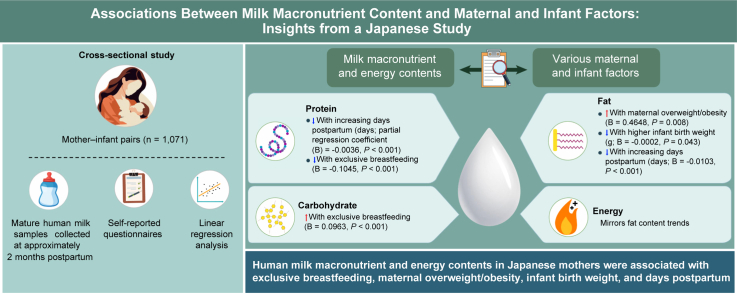

## Introduction

Human milk (HM) is the optimal nutritional source for infants, which contains a diverse array of components essential for growth and development. The WHO recommends exclusive breastfeeding for the first 6 mo of life, followed by continued breastfeeding, aged ≤2 y or older [1]. HM is composed of approximately 87% water, with the remaining 13% consisting primarily of macronutrients including carbohydrates, fats, and proteins [2,3]. These macronutrients are essential sources of energy for infant growth, contributing approximately 50% of total caloric intake from fats, 40% from carbohydrates, and 8% from proteins [2,3]. A recent systematic review indicated a potential association between HM macronutrient composition and infant growth parameters, including weight and length [4]. In addition to supporting growth, HM macronutrients provide essential amino acids and fatty acids involved in immune function, metabolic regulation, and brain development in infants [2,3,5]. These findings underscore the critical role of HM macronutrients in supporting infant development.

HM macronutrient contents vary in response to the infant’s changing needs. Colostrum, the milk secreted immediately after birth, is rich in proteins [6]. As lactation progresses and milk transitions to mature HM, protein content decreases, whereas carbohydrate and fat contents increase [6]. Macronutrient levels also fluctuate based on the time of day (morning, afternoon, or night) and the phase of a feeding session (foremilk compared with hindmilk), with fat content particularly elevated in hindmilk [[7], [8], [9]]. Thus, HM composition undergoes dynamic changes even within the same individual.

In addition to intraindividual variation, HM macronutrient contents vary across individuals and are influenced by maternal health status, lifestyle, and childbirth-related factors. A meta-analysis suggested that HM from mothers with overweight or obesity tends to contain higher concentrations of fat and lactose [10], whereas another meta-analysis of milk from the mothers of preterm infants indicated a higher protein content [6]. Furthermore, factors such as maternal age, infant sex, and cesarean delivery can influence HM macronutrient composition [11,12]. However, some studies have found no significant associations with these factors [[13], [14], [15]]. These inconsistencies in the literature suggest that maternal and infant influences on HM composition may differ across populations, emphasizing the need for country-specific research [[16], [17], [18]]. Despite this need, few comprehensive studies have been conducted among Japanese mothers to investigate the maternal and infant factors affecting HM macronutrient contents.

This study aimed to comprehensively identify factors influencing the macronutrient and energy contents of mature HM in a cross-sectional analysis of a large-scale Japanese cohort. Specifically, we conducted a statistical analysis involving approximately 1000 Japanese mother–infant pairs to examine the wide range of maternal and infant factors associated with mature HM composition.

## Methods

### Participants

This cross-sectional study was conducted during the baseline phase of the Japanese HM Study [19,20]. HM samples were collected at approximately 2 mo postpartum, a period when milk composition is considered mature and relatively stable, thereby providing a representative assessment of the macronutrient and energy contents of mature HM. Details about participant recruitment have been reported previously [19]. Briefly, lactating mothers and their infants were recruited from 73 medical institutions, including 16 hospitals and 57 obstetric clinics across Japan between October 2014 and May 2019. Inclusion criteria were as follows: *1*) healthy lactating mothers, *2*) who had given birth to a single infant, *3*) whose breastfeeding patterns were not disrupted by sample collection, and *4*) who provided samples at ≥15 d postpartum during the baseline survey.

The exclusion criteria were as follows: *1*) positivity for hepatitis B, hepatitis C, human immunodeficiency virus, or human T-cell leukemia virus type 1; *2*) use of medication for underlying conditions; and *3*) non-Japanese mother or partner. Supplemental Figure 1 presents a flowchart outlining participant selection. Among the 1210 lactating mother–infant pairs initially recruited, 139 were excluded: 83 for incomplete responses, 51 for sample collection before 15 d postpartum, and 5 due to a non-Japanese mother or partner. Thus, 1071 mother–infant pairs were included.

#### Items investigated in questionnaires

Data were collected using a self-administered questionnaire designed for the Japanese HM Study [19], which comprised sociodemographic characteristics, maternal anthropometric measurements, medical history, breastfeeding practices, childbirth-related factors, and environmental conditions. This information included maternal age, education, household income, prepregnancy BMI, gestational weight gain, medical history (hyperemesis gravidarum, thyroid disorders, gestational hypertension, and gestational diabetes), smoking status, and season at sample collection. Childbirth data included infant sex, delivery method, parity, gestational age, birth weight, days postpartum, and breastfeeding status at sample collection. To improve data accuracy, mothers were asked to refer to Japan’s Maternal and Child Health Handbook, a nationally standardized record that documents maternal and child health information from pregnancy through early childhood. Breastfeeding status was categorized into exclusive breastfeeding and nonexclusive breastfeeding. Exclusive breastfeeding was defined as infants receiving only HM, with no infant formula given. This definition did not consider the intake of water or supplements. Prepregnancy BMI (kg/m^2^) was categorized as underweight (<18.5), normal weight (18.5 to <25.0), or overweight/obese (≥25.0). The recommended gestational weight gain ranges were defined as 12–15 kg for underweight, 10–13 kg for normal weight, 7–10 kg for overweight, and ≤5 kg for obesity, as established by the Japan Society of Obstetrics and Gynecology [21].

#### Food frequency questionnaire

Maternal nutrient intake was estimated using the brief-type self-administered diet history questionnaire (BDHQ), a shortened version of the self-administered diet history questionnaire (DHQ) developed in Japan [[22], [23], [24]]. The BDHQ is a fixed-portion questionnaire comprising 58 food and beverage items commonly consumed in Japan and includes questions on cooking methods and general eating habits. Dietary intake estimates were calculated using an ad hoc computer algorithm with weighting factors. Among the 58 food and beverage items assessed in the BDHQ, 53 were estimated based on reported intake, frequency of drinking, and fixed-portion size, whereas the remaining 5 items (seasonings, fats and oils, and sugars) were estimated based on cooking methods and general eating habits [22]. Nutrient and energy values were then calculated with reference to the Standard Tables of Food Composition in Japan [25]. The DHQ support center (http://www.ebnjapan.org/) calculated nutrient intake based on food consumption frequency during the previous month. The validity of dietary intake data assessed using the BDHQ was confirmed in a study with 16-d semiweighted dietary records as the reference standard [22]. Although the validity of the BDHQ has not been directly confirmed in lactating females, it has been validated across a wide range of ages, including during pregnancy [[22], [23], [24]]. Furthermore, previous studies have applied the BDHQ to examine associations between dietary patterns and self-reported symptoms in lactating females [26]. Energy-adjusted nutrient intake was derived using the residual method to reduce systematic reporting bias [27]. This study focused on macronutrients (total, animal, and plant protein, total carbohydrate, and total lipid intake).

#### HM collection and analysis

Mothers collected HM samples themselves using a manual breast pump after breastfeeding. Approximately 10–20 mL was transferred into a storage container and stored at −18°C. Samples were collected once daily for 3–7 d and transported via rapid frozen courier. On arrival, samples were stored at −80°C for approximately 1–3 y until analysis. To allow efficient processing, analyses were conducted in multiple batches once adequate sample numbers had been obtained. Before analysis, samples were thawed in a 37–40 °C water bath and pooled per individual to account for day-to-day and diurnal variation, thereby obtaining a representative value for each mother. The samples were subsequently analyzed for macronutrients (true and crude protein, carbohydrate, and fat) and energy using a midinfrared analyzer (MIRIS; Miris Holding). Protein content was reported in 2 forms: with true protein excluding nonprotein nitrogen and crude protein, calculated as total nitrogen × 6.38, which includes nonprotein nitrogen. Quality control procedures for the midinfrared analyzer included duplicate measurements and calibration with manufacturer-provided standard solutions before each batch analysis.

#### Statistical analyses

Descriptive statistics summarized participant characteristics and HM composition. Continuous variables were presented as means and SDs, and categorical variables were presented as counts and percentages. Pearson’s correlation coefficients were used to examine relationships between true protein, crude protein, carbohydrate, fat, and energy contents. All HM macronutrient values were within a physiologically plausible range, the distributions appeared approximately normal based on visual inspection of histograms, and all data were included in the analyses. Univariate linear regression evaluated associations between HM content and maternal or infant factors. Multivariate models were developed using backward stepwise selection, with a removal criterion of *P* > 0.05. In these analyses, HM macronutrient and energy contents were defined as outcomes, and maternal and infant factors were defined as exposures. For the multivariable models, the following variables were initially included in the backward stepwise selection: maternal age, education level, household income, prepregnancy BMI (categorical), dietary intake (animal and plant protein and lipid), gestational weight gain (continuous), medical history (hyperemesis gravidarum, thyroid disorders, gestational hypertension, and gestational diabetes), smoking status, season, infant sex, delivery method, parity, gestational age (continuous), birth weight (continuous), days postpartum, and breastfeeding status. For variables assessed as both continuous and categorical in univariable analyses, either the continuous or categorical form was included to avoid multicollinearity. Variance inflation factors were examined, and variables with a variance inflation factor < 5 were retained in the models. The threshold for statistical significance was set at *P* < 0.05. Analyses were performed using R (version 4.1.1; R Foundation for Statistical Computing) and EZR (version 1.68; Saitama Medical Center, Jichi Medical University) [28].

#### Ethics

The study protocol was approved by the Institutional Review Board of Fukuda Clinic (approval number: IRB20140621-03) and registered in the Japanese Clinical Trials Registry (UMIN000015494). All procedures adhered to the Declaration of Helsinki. All participants provided written informed consent upon enrollment in the Japanese HM Study, including the future use of biological data obtained in the study. Data were anonymized and securely managed to protect confidentiality. Participants could withdraw at any time. Those who completed the questionnaire and provided HM samples received a 3000-Japanese Yen (JPY) gift card (approximately US$27 at the time of the study).

## Results

### Participant characteristics

Table 1 summarizes the characteristics of the 1071 mother–infant pairs included in this study. The mean maternal age was 31 y (SD: 4.4, range: 19–43 y), and 75% had an annual income >4 million JPY. Prepregnancy BMI was within the normal range for 77%, and the mean gestational weight gain was 10.3 kg (SD: 3.6), with 38% falling within the nationally recommended range. Regarding medical history, 19% experienced hyperemesis, and <3% had other conditions. Furthermore, 70% had no history of smoking, and current smokers were rare (<2%). Among the infants, approximately half were males; 12% were delivered by cesarean section; 35% were firstborns; 97% were born full term (37–42 wk), with a mean birth weight of 3078 g (SD: 359); and 4% had low birth weight (<2500 g). The mean infant age at assessment was 54 d (SD: 17.3), and 75% were exclusively breastfed.

**TABLE 1:** Maternal and infant characteristics^1^.

Variable	Number of valid responses	*n* (%)	Mean (SD)
Maternal characteristics			
Age (y)	1071		31.4 (4.4)
Education	1065		
JHS/HS/others		237 (22.3)	
Junior college/technical		452 (42.4)	
4-y college/graduate degree		376 (35.3)	
Household income from all sources, million JPY/y	1030		
<4		274 (26.6)	
4–<8		606 (58.8)	
≥8		150 (14.6)	
Prepregnancy BMI (kg/m^2^)	1061		20.8 (2.7)
<18.5 (Underweight)		170 (16.0)	
18.5–<25.0 (Normal weight)		818 (77.1)	
≥25.0 (Overweight or obesity)		73 (6.9)	
Gestational weight gain (kg)^2^	1055		10.3 (3.6)
Under recommended range		474 (44.9)	
Within recommended range		405 (38.4)	
Over recommended range		176 (16.7)	
Medical history			
Hyperemesis gravidarum	930	172 (18.5)	
Thyroid disease	1069	24 (2.3)	
Hypertension in pregnancy	1069	28 (2.6)	
Gestational diabetes	1069	31 (2.9)	
Smoking status	1067		
Never smoked		740 (69.4)	
Ex-smoker		307 (28.8)	
Current smoker		20 (1.9)	
Nutrient intakes from diet (g/d)			
Protein intake	1063		68.0 (11.2)
Animal protein intake	1063		38.1 (11.8)
Plant protein intake	1063		29.8 (4.4)
Carbohydrate intake	1063		252.2 (28.6)
Lipid intake	1063		58.0 (10.0)
Season at milk sample collection	1071		
Spring (March–May)		270 (25.2)	
Summer (June–August)		245 (22.9)	
Autumn (September–November)		274 (25.6)	
Winter (December–February)		282 (26.3)	
Infant characteristics			
Sex (male)	1071	573 (53.5)	
Cesarean delivery	1066	126 (11.8)	
Parity	1067		
0		376 (35.2)	
1		464 (43.5)	
≥2		227 (21.3)	
Gestational age (wk)	1029		39.0 (1.4)
<37 (Preterm birth)		26 (2.5)	
37–<42 (Term birth)		999 (97.1)	
≥42 (Post-term birth)		4 (0.4)	
Birth weight (g)	1057		3078 (359)
<2500 (Low birth weight)		46 (4.4)	
Days postpartum (d)	1071		54.2 (17.3)
Exclusive breastfeeding at milk sample collection	1069	797 (74.6)	

Abbreviations: HS, high school; JHS, junior high school; JPY, Japanese Yen.

1 Categorical variables are presented as *n* (%), and continuous variables are presented as mean (SD).

2 The recommended ranges for gestational weight gain were defined by the Japan Society of Obstetrics and Gynecology as follows: 12–15 kg for underweight, 10–13 kg for normal weight, 7–10 kg for overweight, and ≤5 kg for obese.

### HM macronutrient and energy contents

Table 2 provides a summary of the HM macronutrient and energy contents. Mean (SD) values for macronutrients and energy were as follows: true protein, 0.95 (0.18) g/100 mL; crude protein, 1.20 (0.23) g/100 mL; carbohydrate, 8.13 (0.32) g/100 mL; fat, 3.77 (1.29) g/100 mL; and energy, 72.6 (12.0) kcal/100 mL.

**TABLE 2:** Macronutrient and energy contents of human milk.

Variable	*n*	Mean (SD)
True protein (g/100 mL)	1071	0.95 (0.19)
Crude protein (g/100 mL)	1071	1.20 (0.23)
Carbohydrate (g/100 mL)	1071	8.13 (0.32)
Fat (g/100 mL)	1071	3.77 (1.29)
Energy (kcal/100 mL)	1071	72.6 (12.0)

**FIGURE 1:**
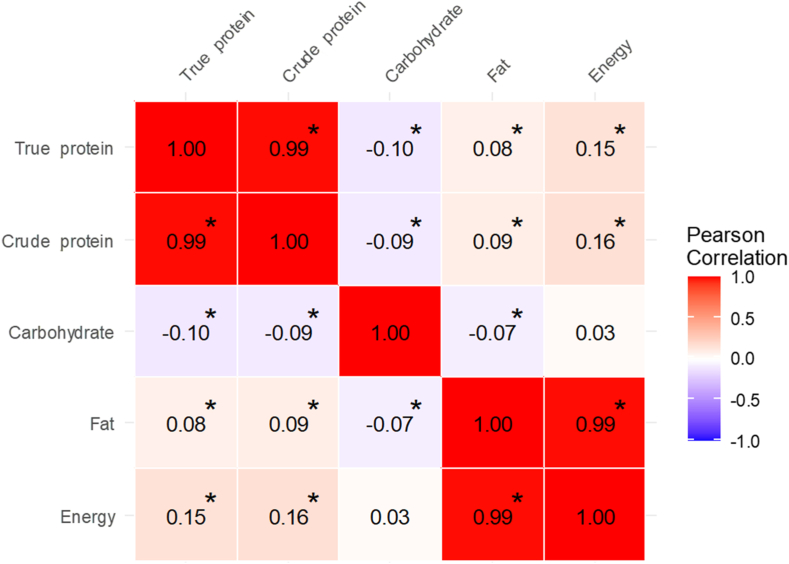
Correlations between true protein, crude protein, carbohydrate, fat, and energy contents of human milk. Coloring reflects the direction and magnitude of Pearson correlation coefficients. ∗Correlation is significant at *P* < 0.05 (*P* values are based on Pearson correlation analysis).

Figure 1 shows correlations among HM true protein, crude protein, carbohydrate, fat, and energy contents. True protein was positively correlated with crude protein, fat, and energy, with a particularly strong correlation with crude protein (*r* = 0.99, *P* < 0.001). Carbohydrate content had significant negative correlations with true protein, crude protein, and fat. Energy content was most strongly correlated with fat (*r* = 0.99, *P* < 0.001).

### Regression analysis

Table 3 presents the univariate linear regression results for factors associated with HM macronutrient and energy contents. True protein content was significantly associated with maternal animal protein intake [g/d; partial regression coefficient (*B*) = 0.0010, *P* = 0.044], parity of 2 compared with 1 (*B* = −0.0317, *P* = 0.014), days postpartum (days; *B* = −0.0038, *P* < 0.001), and exclusive breastfeeding compared with nonexclusive breastfeeding (*B* = −0.1763, *P* < 0.001). Carbohydrate content was significantly associated with a maternal history of thyroid disease (*B* = 0.1395, *P* = 0.033), plant protein intake (g/day; *B* = 0.0048, *P* = 0.031), exclusive breastfeeding compared with nonexclusive breastfeeding (*B* = 0.1598, *P* < 0.001), and fall compared with spring season *(B* = −0.1085, *P* < 0.001). Fat content was significantly associated with prepregnancy overweight or obesity compared with normal weight *(B* = 0.3643, *P* = 0.020), infant birth weight (g; *B* = 0.0002, *P* = 0.045), and days postpartum (days; *B* = −0.0108, *P* < 0.001). Energy content was significantly associated with prepregnancy BMI *(B* = 0.2950, *P* = 0.030), prepregnancy overweight or obesity compared with normal weight (*B* = 3.6073, *P* = 0.014), infant birth weight (g; *B* = 0.0020, *P* = 0.049), and days postpartum (days; *B* = −0.1217, *P* < 0.001). Crude protein content showed a similar pattern to true protein (Supplemental Table 1).

**TABLE 3:** Crude univariate linear regression model examining the associations of milk macronutrients and energy content with maternal and infant characteristics^1^.

Category	Variable	True protein			Carbohydrate			Fat			Energy		
		*B*	SE	*P*	*B*	SE	*P*	*B*	SE	*P*	*B*	SE	*P*
Socio demographic	Age (y)	0.0004	0.0013	0.763	−0.0006	0.0022	0.771	−0.0032	0.0089	0.721	−0.0313	0.0831	0.707
	Education junior college/technical (vs. JHS/HS/others)	0.0025	0.0148	0.865	0.0085	0.0255	0.740	0.0528	0.1035	0.610	0.5310	0.9647	0.582
	Education 4-(y) college/graduate degree (vs. JHS/HS/others)	−0.0029	0.0153	0.849	−0.0071	0.0264	0.789	0.0359	0.1070	0.737	0.2871	0.9976	0.774
	Household income 4–<8 (vs. <4) million JPY/y	0.0018	0.0134	0.893	−0.0256	0.0231	0.269	0.0009	0.0936	0.992	−0.0835	0.8728	0.924
	Household income >8 (vs. <4) million JPY/y	0.0232	0.0186	0.213	0.0248	0.0322	0.443	0.0507	0.1306	0.698	0.6837	1.2177	0.575
Maternal health	Prepregnancy BMI (kg/m^2^)	0.0016	0.0021	0.436	0.0060	0.0036	0.097	0.0285	0.0145	0.051	0.2950	0.1357	0.030^3^
	Prepregnancy underweight (vs. normal weight)^2^	0.0035	0.0156	0.822	−0.0111	0.0268	0.677	−0.1432	0.1081	0.185	−1.3445	1.0081	0.183
	Prepregnancy overweight or obesity (vs. normal weight)^2^	0.0156	0.0226	0.492	0.0363	0.0388	0.351	0.3643	0.1566	0.020^3^	3.6073	1.4609	0.014^3^
	Gestational weight gain (kg)	0.0006	0.0016	0.711	−0.0010	0.0027	0.707	−0.0182	0.0109	0.095	−0.1670	0.1015	0.100
	Gestational weight gain under recommended range (vs. within) 4	−0.0095	0.0125	0.447	0.0216	0.0215	0.316	0.0469	0.0871	0.590	0.4685	0.8121	0.564
	Gestational weight gain over recommended range (vs. within) 4	−0.0086	0.0167	0.604	0.0534	0.0287	0.063	−0.0492	0.1162	0.672	−0.2714	1.0835	0.802
	History of hyperemesis gravidarum — yes (vs. no)	0.0099	0.0158	0.532	0.0309	0.0258	0.231	−0.0756	0.1086	0.486	−0.5196	1.0158	0.609
	History of thyroid disease—yes (vs. no)	0.0288	0.0383	0.451	−0.1395	0.0654	0.033^3^	0.1165	0.2660	0.661	0.6560	2.4816	0.792
	History of hypertension in pregnancy—yes (vs. no)	0.0454	0.0355	0.200	−0.0039	0.0608	0.948	0.1521	0.2467	0.538	1.6363	2.3014	0.477
	History of gestational diabetes—yes (vs. no)	0.0441	0.0338	0.191	−0.0596	0.0578	0.303	0.2469	0.2347	0.293	2.2733	2.1898	0.299
Maternal diet	Protein intake (g/d)	0.0010	0.0005	0.051	0.0002	0.0009	0.790	−0.0014	0.0035	0.692	−0.0066	0.0329	0.842
	Animal protein intake (g/d)	0.0010	0.0005	0.044^3^	−0.0005	0.0008	0.581	−0.0029	0.0034	0.392	−0.0230	0.0313	0.463
	Plant protein intake (g/d)	−0.0005	0.0013	0.693	0.0048	0.0022	0.031^3^	0.0116	0.0090	0.199	0.1225	0.0840	0.145
	Carbohydrate intake (g/d)	−0.0002	0.0002	0.236	0.0004	0.0003	0.242	0.0003	0.0014	0.834	0.0028	0.0129	0.831
	Lipid intake (g/d)	0.0004	0.0006	0.536	−0.0017	0.0010	0.081	−0.0005	0.0040	0.898	−0.0087	0.0369	0.813
Infant and lactation	Sex— male (vs. female)	−0.0072	0.0113	0.523	0.0229	0.0194	0.240	−0.0315	0.0789	0.690	−0.2404	0.7362	0.744
	Cesarean delivery (vs. vaginal)	0.0222	0.0176	0.208	0.0376	0.0301	0.212	−0.1474	0.1224	0.229	−1.0872	1.1417	0.341
	Parity 2 (vs. 1)	−0.0317	0.0128	0.014^3^	0.0197	0.0220	0.371	−0.0841	0.0894	0.347	−0.8882	0.8339	0.287
	Parity ≥3 (vs. 1)	0.0139	0.0155	0.372	−0.0061	0.0267	0.818	−0.1179	0.1083	0.277	−1.0601	1.0102	0.294
	Gestational age (wk)	−0.0052	0.0042	0.222	−0.0036	0.0073	0.623	−0.0126	0.0293	0.667	−0.1581	0.2741	0.564
	Gestational age <37 (vs. ≥37) (wk)	0.0165	0.0368	0.653	−0.0601	0.0636	0.345	−0.0158	0.2546	0.951	−0.3067	2.3777	0.897
	Birth weight (g)	−0.00002	0.00002	0.257	0.00002	0.00003	0.386	−0.0002	0.0001	0.045^3^	−0.0020	0.0010	0.049^3^
	Birth weight <2500 (vs. ≥2500) (g)	0.0106	0.0279	0.705	−0.0224	0.0478	0.639	−0.0641	0.1944	0.742	−0.6536	1.8149	0.719
	Days postpartum (d)	−0.0038	0.0003	<0.001^3^	−0.0002	0.0006	0.763	−0.0108	0.0022	<0.001^3^	−0.1217	0.0209	<0.001^3^
	Exclusive breastfeeding (vs. nonexclusive breastfeeding)	−0.1109	0.0126	<0.001^3^	0.1120	0.0219	<0.001^3^	0.1045	0.0901	0.247	0.8345	0.8415	0.322
Environment	Ex-smoker (vs. never smoked)	−0.0113	0.0126	0.368	−0.0139	0.0216	0.519	0.1307	0.0875	0.135	1.0949	0.8165	0.180
	Current smoker (vs. never smoked)	−0.0026	0.0420	0.951	−0.0369	0.0720	0.609	0.2332	0.2921	0.425	1.9686	2.7256	0.470
	Summer (vs. spring)	−0.0069	0.0163	0.672	−0.0435	0.0278	0.118	−0.1501	0.1136	0.187	−1.6081	1.0596	0.129
	Fall (vs. spring)	−0.0245	0.0159	0.123	−0.1085	0.0270	<0.001^3^	−0.0923	0.1104	0.403	−1.4598	1.0298	0.157
	Winter (vs. spring)	−0.0295	0.0158	0.061	−0.0406	0.0268	0.131	−0.0040	0.1097	0.971	−0.3942	1.0225	0.700

Abbreviations: *B*, partial regression coefficient; HS, high school; JHS, junior high school; JPY, Japanese Yen.

1 Crude univariate linear regression model with macronutrient and energy contents of human milk as the dependent variables, and maternal and infant characteristics as independent variables. *P* values are based on these univariate linear regression models.

2 Maternal prepregnancy BMI (kg/m^2^) was classified as underweight (<18.5), normal weight (18.5–<25.0), or overweight or obesity (≥25.0).

3 Association is significant at *P* < 0.05.

4 The recommended ranges for gestational weight gain were defined as 12–15 kg for underweight, 10–13 kg for normal weight, 7–10 kg for overweight, and ≤5 kg for obesity, in accordance with guidelines from the Japan Society of Obstetrics and Gynecology.

Table 4 lists the results of the stepwise multivariate linear regression model performed to identify the determinants for HM macronutrient and energy contents. True protein content was significantly associated with days postpartum (days; *B* = −0.0036, *P* < 0.001) and exclusive breastfeeding compared with nonexclusive breastfeeding *(B* = −0.1045, *P* < 0.001). Carbohydrate content was significantly associated with exclusive breastfeeding compared with nonexclusive breastfeeding *(B* = 0.0963, *P* < 0.001). Fat content was significantly associated with prepregnancy overweight or obesity compared with normal weight (*B* = 0.4648, *P* = 0.008), infant birth weight (g; *B* = −0.0002, *P* = 0.043), and days postpartum (days; *B* = −0.0103, *P* < 0.001). Energy content was significantly associated with prepregnancy overweight or obesity compared with normal weight *(B* = 4.5540, *P* = 0.006), infant birth weight (g; *B* = −0.0022, *P* = 0.047), and days postpartum (days; *B* = −0.1170, *P* < 0.001). Crude protein showed a similar pattern to true protein content (Supplemental Table 2).

**TABLE 4:** Stepwise multivariable regression model examining the associations of milk macronutrients and energy contents with maternal and infant characteristics^1^.

Variable	True protein (*R*^2^ = 0.195)			Carbohydrate (*R*^2^ = 0.018)			Fat (*R*^2^ = 0.027)			Energy (*R*^2^ = 0.038)		
	*B*	SE	*P*	*B*	SE	*P*	*B*	SE	*P*	*B*	SE	*P*
Prepregnancy underweight (vs. normal weight) 2	—	—	—	—	—	—	−0.0458	0.1230	0.710	−0.4493	1.1450	0.695
Prepregnancy overweight or obesity (vs. normal weight) 2	—	—	—	—	—	—	0.4648	0.1761	0.008^3^	4.5540	1.6388	0.006^3^
Birth weight (g)	—	—	—	—	—	—	−0.0002	0.0001	0.043^3^	−0.0022	0.0011	0.047^3^
Days postpartum (d)	−0.0036	0.0003	<0.001^3^	—	—	—	−0.0101	0.0025	<0.001^3^	−0.1170	0.0230	<0.001^3^
Exclusive breastfeeding (vs. nonexclusive breastfeeding)	−0.1045	0.0132	<0.001^3^	0.0963	0.0242	<0.001^3^	—	—	—	—	—	—

Abbreviation: *B*, partial regression coefficient.

1 Stepwise multivariable linear regression model with macronutrient and energy contents of human milk as the dependent variables, and maternal and infant characteristics as independent variables. *P* values are based on the final multivariable linear regression model after backward stepwise selection, adjusted for all covariates retained.

2 Maternal prepregnancy BMI (kg/m^2^) was classified as underweight (<18.5), normal weight (18.5–<25.0), or overweight or obese (≥25.0).

3 Association is significant at *P* < 0.05.

## Discussion

This study suggests that the macronutrient and energy contents of mature HM in Japanese mothers were associated with various maternal and infant factors. True and crude protein contents were negatively associated with days postpartum and exclusive breastfeeding, whereas carbohydrate content was positively associated with exclusive breastfeeding. Fat and energy contents were positively associated with prepregnancy overweight or obesity and negatively associated with infant birth weight and days postpartum. The crude protein and energy contents followed patterns similar to those of true protein and fat, respectively. These similarities are likely attributable to the strong correlations between these respective components. Additionally, the mean macronutrient and energy contents of HM found in this study were comparable with those reported in previous studies using the same midinfrared HM analyzer [[29], [30], [31]].

Days postpartum was negatively associated with HM protein, fat, and energy contents; no association was observed for carbohydrate content. These results are consistent with previous studies on early-stage mature HM. In our study, HM samples were collected at an average of 2 mo postpartum, representing early mature HM. Protein content is known to decline from colostrum to mature HM and continues to decrease gradually, which is a well-established pattern in prior studies [6,32,33]. HM fat content increases from colostrum to mature HM [6,34], although findings on mature HM collected during early lactation are mixed; some report a decline, and others note no significant change [[34], [35], [36]]. Carbohydrate content has consistently been reported to increase from colostrum to mature HM and stabilizing thereafter, aligning with our findings [6,32,37].

Prepregnancy overweight and obesity were positively associated with HM fat and energy contents. This finding aligns with a meta-analysis demonstrating a similar pattern across countries [10]. To our knowledge, this was the first report of such associations in Japanese mothers. Physiologically, this may reflect dyslipidemia, elevated triglycerides, and increased insulin concentrations, all common among individuals with higher BMI and fat mass [38,39]. Additionally, elevated HM fat content has been linked to increased infant body fat [31], and maternal obesity has been associated with childhood obesity in meta-analyses [40,41]. Therefore, HM fat content may partially mediate the intergenerational link in obesity.

Exclusive breastfeeding was negatively associated with protein content and positively associated with carbohydrate content. Few studies have explored this relationship. A longitudinal study in China found exclusive breastfeeding was linked to reduced protein and lactose contents at day 10 postpartum and to reduced lactose at day 60 postpartum [42]. This differs from our carbohydrate content findings and may reflect population differences, particularly in cesarean section rates (47% in China compared with 12% in our study) [42]. Although our data showed no link between cesarean section and HM carbohydrate content, other studies involving higher cesarean section rates have reported such associations [12,43], likely due to hormonal changes affecting HM secretion. Furthermore, HM volume has been shown to be negatively associated with protein content and positively associated with lactose content [44,45]. This suggests that mothers with higher HM volume may have nutrient profiles similar to those practicing exclusive breastfeeding, as observed in our study. Perceived insufficient milk supply is a common reason for discontinuing exclusive breastfeeding [[46], [47], [48]]. In our supplemental survey, over half of nonexclusively breastfeeding mothers cited insufficient milk production. Thus, the observed macronutrient changes may reflect variations in milk volume. However, evidence linking perceived milk insufficiency to actual volume is limited, and our study did not assess milk volume, warranting further investigation.

Infant birth weight was negatively associated with HM fat content. A study from Greece reported a similar association across all HM stages [49]. In contrast, a Korean study linked HM fat content to infant height but not weight [12]. Although results remain inconsistent, HM fat may play a role in regulating infant weight gain in response to birth weight.

Changes in HM macronutrient and energy contents can impact infant growth and health in both the short and long term. Prior studies have linked HM composition to infant weight, fat percentage, and weight-for-length *Z*-scores in the first 6 mo [31,50,51]. Moreover, rapid infant weight gain is associated with higher fat mass and metabolic syndrome risk in early adulthood [[52], [53], [54]]. Identifying determinants of HM composition, as partially achieved in this study, may help to mitigate future health risks.

The strength of this study lies in its comprehensive analysis of diverse maternal and infant factors in a large cohort. To the best of our knowledge, few studies have examined the associations between maternal and infant factors and HM macronutrient contents using such a large scale and from a similarly multifaceted perspective, making this a pioneering investigation. Moreover, the study offers valuable insights to fill a knowledge gap specific to Japanese populations.

However, this study has a few limitations. First, the study population was skewed toward healthy mothers and infants, necessitating caution in generalizing the findings. Mothers and infants with delivery complications were excluded, and the underrepresentation of preterm or low birth weight infants may have led to underestimation of the influence of these factors [20]. Second, the methods of HM sample collection and storage may have influenced variation in composition. HM samples were not collected throughout the entire day, which may have introduced random error due to the feeding session stage and diurnal variation [[7], [8], [9]]. To standardize collection while minimizing breastfeeding disruption, HM samples were collected after breastfeeding, likely consisting primarily of mid- or hindmilk. Additionally, samples were collected once daily for 3–7 d and pooled per individual, which likely reduced the impact of diurnal variation. Priority was placed on maximizing sample acquisition and minimizing interference with breastfeeding rather than enforcing strict standardization of collection conditions. Moreover, HM samples were stored at −80 °C for approximately 1–3 y before analysis because analyses were conducted in multiple batches once adequate sample numbers had been obtained. Although differences in storage duration may have affected HM composition, previous studies have shown that storage at –70°C has minimal impact on the macronutrient composition of HM, suggesting that any effect was likely small [55]. Third, maternal and infant factors were obtained from a self-administered questionnaire, which may have introduced misreporting and limited the generalizability of our findings. However, the anthropometric data were recorded based on Japan’s Maternal and Child Health Handbook, which documents maternal and child health information from pregnancy through early childhood, thereby minimizing recall bias. Finally, because this was a cross-sectional study, causality could not be established. Additionally, as HM composition was assessed at only a single stage of lactation (around 2 mo postpartum), the natural trajectory of macronutrient changes across other stages could not be evaluated, and the associated factors might also differ at different stages. However, this stage was selected because HM composition is considered mature and relatively stable, and most infants have not yet started complementary feeding, thereby reducing variability in maternal and infant factors and allowing for a more consistent assessment of their associations with HM composition.

In conclusion, HM true and crude protein contents were negatively associated with days postpartum and exclusive breastfeeding, whereas HM carbohydrate content was positively associated with exclusive breastfeeding. In contrast, HM fat and energy contents were positively associated with maternal overweight or obesity and negatively associated with birth weight and days postpartum. These findings suggest that the macronutrient and energy content of mature HM in Japanese mothers is associated with maternal and infant physical characteristics, breastfeeding practices, and postpartum duration, but not with sociodemographic, nutritional, or environmental factors. This aligns with previous reports showing that HM macronutrient composition remains relatively stable even under economic hardship and nutritional deficiency [56,57]. These results may support breastfeeding promotion efforts in Japan and inform international comparative studies on HM composition. Longitudinal research is warranted to explore the biological effects of the identified macronutrient variations on the growth and health of breastfed infants.

## Author contributions

The authors’ responsibilities were as follows – KNojiri: conceived and designed the project and study and prepared the original draft; KNojiri, SH, TT, YT: organized the sociodemographic data and conducted the analysis of human milk samples; KNojiri, KNomura: interpreted the data; KNojiri, SH, KNomura: had full data access, conducted statistical analyses, and assumed responsibility for data integrity and accuracy; SH: coordinated the project and the Japanese Human Milk Study Cohort; KNomura, SH: supervised the project; KNomura, SH, TY: reviewed and edited the manuscript; and all authors: read and approved the final manuscript.

## Data availability

Data described in the manuscript, code book, and analytic code will be made available upon request pending.

## Funding

This work was supported by Bean Stalk Snow Co., Ltd., and Megmilk Snow Brand Co., Ltd.

## Conflict of interest

KNojiri, TT, YT, TY, and SH were employees of Bean Stalk Snow. Bean Stalk Snow manufactures and sells dietary supplements for perinatal females. Megmilk Snow Brand manufactures and sells dairy foods. KNomura reports no conflicts of interest. These organizations did not conduct any violations, interferences, or substantial influences on the scientific observations and conclusions in this study.
