# Supplementary material for: Human Milk Macronutrient and Energy Contents Are Associated with Maternal and Infant Factors: A Cross-Sectional Analysis of Data from the Japanese Human Milk Study Cohort
Source: Curr Dev Nutr. 2025 Oct 17;9(11):107579. doi: 10.1016/j.cdnut.2025.107579 (PMC12651646; doi:10.1016/j.cdnut.2025.107579)
Supplement: multimedia component 1 [file mmc1.docx]

**Supplemental Figure 1.** Participant flowchart

Participants recruited in the Japanese Human Milk Study (n = 1,210) (n=1,210)

Participants who did not meet the inclusion/exclusion criteria

Excluded (n = 139)

Did not provide a valid response (n = 83)

Did not provide human milk samples after 15 days postpartum during the baseline period (n = 51)

Non-Japanese ethnicity (n = 5)

Eligible participants (n = 1,071)

**Supplemental Table 1.** Crude univariate linear regression model examining the associations of milk crude and true protein content with maternal and infant characteristics.^1^

| **Category** | **Variable** | **Crude protein** | | | **True protein** | | |
| --- | --- | --- | --- | --- | --- | --- | --- |
|  |  | B | SE | *P* | B | SE | *P* |
| Socio demographic | Age, year | 0.0002 | 0.0016 | 0.925 | 0.0004 | 0.0013 | 0.763 |
|  | Education junior college/technical (vs.JHS/HS/others) | 0.0021 | 0.0184 | 0.911 | 0.0025 | 0.0148 | 0.865 |
|  | Education 4-year college/graduate degree (vs. JHS/HS/others) | -0.0038 | 0.0190 | 0.842 | -0.0029 | 0.0153 | 0.849 |
|  | House hold income 4–<8 (vs. <4), million JPY/year | 0.0023 | 0.0166 | 0.889 | 0.0018 | 0.0134 | 0.893 |
|  | House hold income >8 (vs. <4), million JPY/year | 0.0263 | 0.0232 | 0.257 | 0.0232 | 0.0186 | 0.213 |
| Maternal health | Pre-pregnancy BMI, kg/m^2^ | 0.0018 | 0.0026 | 0.490 | 0.0016 | 0.0021 | 0.436 |
|  | Pre-pregnancy underweight (vs. normal weight)^2^ | 0.0056 | 0.0194 | 0.773 | 0.0035 | 0.0156 | 0.822 |
|  | Pre-pregnancy overweight or obesity (vs. normal weight) ^2^ | 0.0210 | 0.0282 | 0.456 | 0.0156 | 0.0226 | 0.492 |
|  | Gestational weight gain, kg | 0.0011 | 0.0019 | 0.562 | 0.0006 | 0.0016 | 0.711 |
|  | Gestational weight gain under recommended range (vs. within) ^3^ | -0.0118 | 0.0156 | 0.447 | -0.0095 | 0.0125 | 0.447 |
|  | Gestational weight gain over recommended range (vs. within) ^3^ | -0.0069 | 0.0208 | 0.740 | -0.0086 | 0.0167 | 0.604 |
|  | History of hyperemesis gravidarum - yes (vs. no) | 0.0129 | 0.0198 | 0.516 | 0.0099 | 0.0158 | 0.532 |
|  | History of thyroid disease - yes (vs. no) | 0.0309 | 0.0477 | 0.516 | 0.0288 | 0.0383 | 0.451 |
|  | History of hypertension in pregnancy- yes (vs. no) | 0.0557 | 0.0442 | 0.208 | 0.0454 | 0.0355 | 0.200 |
|  | History of gestational diabetes - yes (vs. no) | 0.0517 | 0.0421 | 0.219 | 0.0441 | 0.0338 | 0.191 |
| Maternal diet | Protein intake, g/day | 0.0012 | 0.0006 | 0.052 | 0.0010 | 0.0005 | 0.051 |
|  | Animal protein intake, g/day | 0.0012 | 0.0006 | 0.040* | 0.0010 | 0.0005 | 0.044* |
|  | Plant protein intake, g/day | -0.0009 | 0.0016 | 0.590 | -0.0005 | 0.0013 | 0.693 |
|  | Carbohydrate intake, g/day | -0.0003 | 0.0002 | 0.163 | -0.0002 | 0.0002 | 0.236 |
|  | Lipid intake, g/day | 0.0006 | 0.0007 | 0.371 | 0.0004 | 0.0006 | 0.536 |
| Infant and lactation | Sex - male (vs. female) | -0.0093 | 0.0141 | 0.512 | -0.0072 | 0.0113 | 0.523 |
|  | Cesarean delivery (vs. vaginal) | 0.0286 | 0.0219 | 0.192 | 0.0222 | 0.0176 | 0.208 |
|  | Parity 2 (vs. 1) | -0.0430 | 0.0160 | 0.007* | -0.0317 | 0.0128 | 0.014* |
|  | Parity ≥3 (vs. 1) | 0.0125 | 0.0193 | 0.518 | 0.0139 | 0.0155 | 0.372 |
|  | Gestational age, week | -0.0061 | 0.0053 | 0.248 | -0.0052 | 0.0042 | 0.222 |
|  | Gestational age <37 (vs. ≥37), week | 0.0181 | 0.0459 | 0.693 | 0.0165 | 0.0368 | 0.653 |
|  | Birth weight, g | -0.00002 | 0.00002 | 0.349 | -0.00002 | 0.00002 | 0.257 |
|  | Birth weight <2,500 (vs. ≥ 2,500), g | 0.0066 | 0.0348 | 0.850 | 0.0106 | 0.0279 | 0.705 |
|  | Days postpartum, day | -0.0048 | 0.0004 | <0.001* | -0.0038 | 0.0003 | <0.001* |
|  | Exclusive breastfeeding (vs. non-exclusive breastfeeding) | -0.1318 | 0.0157 | <0.001* | -0.1109 | 0.0126 | <0.001* |
| Environment | Ex-smoker (vs. never smoked) | -0.0133 | 0.0157 | 0.395 | -0.0113 | 0.0126 | 0.368 |
|  | Current smoker (vs. never smoked) | -0.0092 | 0.0523 | 0.860 | -0.0026 | 0.0420 | 0.951 |
|  | Summer （vs. spring） | -0.0103 | 0.0203 | 0.612 | -0.0069 | 0.0163 | 0.672 |
|  | Fall （vs. spring） | -0.0390 | 0.0197 | 0.048* | -0.0245 | 0.0159 | 0.123 |
|  | Winter （vs. spring） | -0.0444 | 0.0196 | 0.024* | -0.0295 | 0.0158 | 0.061 |

^1^Crude univariate linear regression model with protein contents of human milk as the dependent variables, and maternal and infant characteristics as independent variables. P-values are based on these univariate linear regression models. HS, high school; JHS, junior high school; JPY, Japanese yen; BMI, body mass index; B, partial regression coefficient; SE, standard error.

^2^Maternal pre-pregnancy BMI (kg/m^2^) was classified as underweight (<18.5), normal weight (18.5–<25.0), or overweight/obesity (≥25.0).

^3^The recommended ranges for gestational weight gain were defined as 12–15 kg for underweight, 10–13 kg for normal weight, 7–10 kg for overweight, and ≤5 kg for obesity, in accordance with guidelines from the Japan Society of Obstetrics and Gynecology.

*Association is significant at *P* < 0.05.

**Supplemental Table 2.** Stepwise multivariable linear regression model examining the associations of milk crude and true protein contents with maternal and infant characteristics.^1^

| **Variable** | **Crude protein**  **(R^2^ = 0.192)** | | | **True protein**  **(R^2^ = 0.195)** | | |
| --- | --- | --- | --- | --- | --- | --- |
|  | B | SE | *P* | B | SE | *P* |
| Days postpartum, day | -0.0045 | 0.0004 | <0.001 | -0.0036 | 0.0003 | <0.001 |
| Exclusive breastfeeding (vs. non-exclusive breastfeeding) | -0.1291 | 0.0165 | <0.001 | -0.1045 | 0.0132 | <0.001 |

^1^Stepwise multivariable linear regression model with protein contents of human milk as the dependent variables, and maternal and infant characteristics as independent variables. P-values are based on the final multivariable linear regression model after backward stepwise selection, adjusted for all covariates retained. B, partial regression coefficient; SE, standard error.

*Association is significant at *P* < 0.05.
